# Prevalence of risk factors for non-communicable diseases in the Mekong Delta, Vietnam: results from a STEPS survey

**DOI:** 10.1186/1471-2458-9-291

**Published:** 2009-08-12

**Authors:** Luc H Pham, Thuy B Au, Leigh Blizzard, Nhan B Truong, Michael D Schmidt, Robert H Granger, Terence Dwyer

**Affiliations:** 1 Faculty of Public Health, Can Tho University of Medicine and Pharmacy, Can Tho, Vietnam; 2 Menzies Research Institute, University of Tasmania, Hobart, Australia; 3 Department of Kinesiology, University of Georgia, Athens, USA; 4 Department of Plastic and Reconstructive Surgery, Royal Hobart Hospital, Hobart, Australia; 5 Murdoch Children's Research Institute, Royal Children's Hospital, Melbourne, Australia

## Abstract

**Background:**

Despite the increasing burden of non-communicable diseases (NCD) in Vietnam, information on the prevalence of preventable risk factors for NCD is restricted to the main urban centres of Ha Noi, and Ho Chi Minh City (HCMC). This population-based survey aimed to describe the prevalence of risk factors for NCD in a rural Vietnamese sample.

**Methods:**

This survey was conducted using the WHO "STEPwise approach to surveillance of non-communicable diseases" (STEPS) methodology. Participants (n = 1978) were residents of the Mekong Delta region selected by multi-stage sampling. Standardised international protocols were used to measure behavioural risk factors (smoking, alcohol consumption, fruit and vegetable consumption, physical activity), physical characteristics (weight, height, waist and hip circumferences, blood pressure – BP), fasting blood glucose (BG) and total cholesterol (TC). Data were analysed using complex survey analysis methods.

**Results:**

In this sample, 8.8% of men and 12.6% of women were overweight (body mass index (BMI) ≥ 25 kg/m^2^) and 2.3% of men and 1.5% of women were obese (BMI ≥ 30 kg/m^2^). The prevalence of hypertension (systolic BP ≥ 140 mmHg and/or diastolic BP ≥ 90 mmHg, or taking medication for hypertension) was 27.3% for men and 16.2% for women. There were 1.0% of men and 1.1% of women with raised BG (defined as capillary whole BG of at least 6.1 mmol/L).

**Conclusion:**

We provide the first NCD risk factor profile of people living in the Mekong Delta of Vietnam using standardised methodology. Our findings for this predominantly rural sample differ from previous studies conducted in Ha Noi and HCMC, and suggest that it is inappropriate to generalise findings from the big-city surveys to the other 80% of the population.

## Background

Despite the increasing burden of non-communicable diseases (NCD) in Vietnam [1,2], information on the prevalence of preventable risk factors for NCD is restricted to the main urban centres of Ha Noi [3-6], and Ho Chi Minh City (HCMC) [7-10]. Taken together, these studies paint an incomplete picture of the NCD risk factor profile of the Vietnamese people. In particular, there is a critical lack of information for the 80% of the population living outside the industrialised areas in and around Ha Noi in the North and HCMC in the South. With the exception of a study conducted in Bavi [5,6], a poor district of Ha Noi, little is known about the NCD risk factor profiles of people living in rural areas of Vietnam.

Home to 21 percent of the country's population, the Mekong Delta – literally the "nine dragon river delta" – is the far southern region of Vietnam. The tributaries of the Mekong River act as a transport network and deposit alluvium, increasing the fertility of the soil that produces abundant harvests of rice and other crops [11]. The river system is also a significant source of food to the population via the fish it supplies. While the Mekong Delta is the main food production area of the country, health services in this region (assessed as number of hospital beds and number of health personnel per 100,000 people) are below the country average [12]. There are no studies apart from our own work that investigate the prevalence of NCD risk factors among residents of the Mekong Delta. Presenting the NCD risk profile for this population would provide the first systemic information of its type to compare with that of other regions and to serve as a baseline for future studies. Moreover, this information will help local authorities to prioritise the health service and health promotion interventions in the region.

This study aimed to describe the prevalence of risk factors for NCD in a rural Vietnamese sample from the Mekong Delta using standardised survey methodology developed by the World Health Organization (WHO) – the STEPwise approach to surveillance of non-communicable diseases (STEPS) [13]. In addition, we compare estimates for men and women in this sample, and our results to those of previous surveys conducted in the two big cities and discuss possible explanations for the differences found.

## Methods

### Subjects and sampling

This population-based survey was conducted among 25–64 year old residents of Can Tho in the Mekong Delta, Vietnam. Eligible subjects were selected by multistage sampling with age, sex, and urban/rural stratification. In brief, the sampling process was as follows. At the first stage, a sample of eight urban- and eight rural-classified communes was selected with probability proportional to size and with replacement. The second-stage sampling units were health volunteers who are responsible for providing basic health services for residents living in their local area. The health volunteers maintain and update the lists of these people regularly. Collectively the health volunteers cover all households in each commune. One health volunteer was chosen from each selected commune with probability proportional to size of the population for which they were responsible and with replacement. Health volunteers who were responsible for only a small number of households were combined prior to sampling. At the third stage, persons were selected from the list of each selected health volunteer, with stratification for age (we sought equal numbers in the four age categories 25–34 years, 35–44 years, 45–54 years, and 55–64 years) and sex (we sought equal numbers of men and women). The target number of participants in each commune was 125. People who were institutionalized at the time of data collection were excluded. The sample of eligible subjects consisted of 2683 persons of whom 73.7% (1978/2683) participated in this survey.

Informed consent was obtained from participants. Those who could not sign provided verbal consent. The study was approved by the Ethics Committee of Can Tho University of Medicine and Pharmacy. Data collection was carried out from July to November 2005.

### Measurements

Measurements by questionnaire consisted of demographic characteristics, socio-economic factors, and four behavioural risk factors (smoking, alcohol consumption, fruit and vegetable consumption and physical activity). The questionnaire was modified with expanded and optional questions to suit local needs. Extended questions were questions in the STEPS instrument modified by adding locally relevant response options (that described types of work specific to the local area, for example). Optional questions were new questions added to the instrument because they were deemed locally important (in relation to passive smoking, for example). All the modifications were done in accordance with the WHO STEPS manual [13]. The questionnaire was translated into Vietnamese and back-translated by independent translators to ensure the appropriate meaning of each item was retained.

Physical measurements included weight (in bare feet without heavy clothing measured using Seca 767 digital scales), height (in bare feet without headwear measured using a Seca 220 stadiometer), waist circumference (at the narrowest point between the lower costal border and the iliac crest measured using a constant tension tape), hip circumference (at the greatest posterior protuberance of the buttocks measured using a constant tension tape), and blood pressure (at the midpoint of the right arm after participants had rested for at least five minutes measured using an Omron T9P digital automatic blood pressure monitor). Two blood pressure readings were obtained for all participants. A third reading was taken if there was a difference of more than 25 mmHg for systolic blood pressure (SBP) or 15 mmHg for diastolic blood pressure (DBP) between the first two readings. The mean of all measures was used.

Biochemical measures included fasting total cholesterol (TC) and fasting blood glucose (BG) measured in capillary blood using a Roche Diagnostics Accutrend Glucometer.

Data collection staff were medical doctors, laboratory technicians and medical students. They underwent intensive training and supervision provided by the Menzies Research Institute. A pilot study was conducted to test survey instruments and procedures before actual data collection. Questionnaires were administered by face-to-face interviews. All measurements were performed in accordance with the WHO STEPS protocols [13] at a clinic set up at 16 different field testing sites.

### Statistical methods

Data were coded and presented according to WHO guidelines [13]. Hours of physical activity of moderate and vigorous intensities were weighted by their Metabolic Equivalent Task (MET) values provided in the WHO guidelines (moderate activity is assigned a MET of 4 and vigorous activity is assigned a MET of 8). Analyses were performed using STATA software version 9.2. Complex survey analysis methods were used to estimate the prevalence of study factors taking into account the sampling design and the sampling weight of each participant. A sampling weight for each participant was calculated as the inverse of the probability of selection of that particular participant taking into account each stage of the sampling process. The age structure of the Vietnamese population from the 1999 census [14] was used to estimate the age-standardised prevalence of hypertension. In regression analyses, we investigated whether differences between men and women in change in SBP, BG and TC with age could be explained by differences in the four behavioural risk factors or BMI.

## Results

The sample was dominated by persons of Vietnamese ethnicity with Chinese and Khmer in the minority. The majority of participants (particularly women) did not complete secondary school to grade 9, and most were self-employed. The most common occupation (particularly among men) was farming. Selected characteristics of the participants are shown in Table 1.

**Table 1:** Characteristics of study participants in Can Tho, 2005.

	Men (N = 911)		Women (N = 1067)		*p*
			
	%	n	%	n	
Age					
25–34	17.2	157	18.8	201	
35–44	26.9	245	27.4	292	
45–54	29.6	270	28.2	301	
55–64	26.2	239	25.6	273	*0.003*
					
Ethnicity					
Vietnamese	92.2	839	91.0	970	
Chinese	1.5	14	2.3	24	
Khmer	6.2	56	6.8	72	
Others	0.1	1	0	0	*0.059*
					
Education completed					
< Primary school	38.0	346	55.6	593	
Primary school	27.5	250	21.1	225	
Secondary school	17.8	162	11.9	127	
High school*	10.9	99	7.2	77	
College/University^†^	5.8	53	4.1	44	*0.002*
					
Employment status					
Employed	13.1	119	7.4	79	
Self-employed	62.4	568	50.2	535	
Non-paid/student	0.6	5	0.8	8	
Homemaker	0.8	7	25.5	272	
Retired/unemployed^‡^	7.0	64	4.7	50	
Unstably employed^§^	16.2	147	11.4	122	*<0.001*
					
Occupation					
Farmers	35.9	362	24.1	294	
Industrial workers	9.4	73	2.6	16	
Clerks	9.6	66	7.2	58	
Traders	13.8	107	23.7	227	
Homemakers	0.0	0	22.4	257	
Others	31.3	302	20.0	214	*<0.001*

* High school or equivalent (technical school).

^† ^College or university degree or higher.

^‡ ^Including 12 men and 6 women who were disabled.

^§ ^People who did physical work and got paid on a daily basis.

There were one man and one woman who provided only information on age and sex.

Table 2 presents prevalence of behavioural risk factors for men and women. Reflecting cultural practice, the prevalence of smoking and alcohol consumption were much higher in men than in women. Additionally, 80.8% (730/910) of men and 50.6% (526/1066) of women reported being exposed daily to tobacco smoke either from themselves or someone else. The average time spent doing moderate and/or vigorous physical activities was 20.47 (95%CI: 15.96–24.98) hours/week for men and 16.27 (95%CI: 13.73–18.80) hours/week for women. The average time spent in sedentary activity was 3.83 (95%CI: 3.26–4.40) hours/day for men and 3.37 (95%CI: 2.81–3.92) hours/day for women.

**Table 2:** Prevalence of behavioural risk factors for NCD* in Can Tho, 2005.

	Men (N = 910)		Women (N = 1066)	
		
	% ± SE^†^	n	% ± SE^†^	n
Smoking				
Current smoker	67.8 ± 1.8	631	1.1 ± 0.5	16
Daily smoker	63.1 ± 2.2	594	0.6 ± 0.3	10
Non-daily smoker	4.6 ± 0.9	37	0.4 ± 0.2	6
Ex-smoker	13.0 ± 1.1	130	0.1 ± 0.1	3
Never smoker	19.2 ± 1.6	149	98.8 ± 0.6	1047
				
Alcohol consumption				
Ever consume alcohol	87.2 ± 1.7	794	11.6 ± 1.0	127
Consume last 12 months^‡^	80.9 ± 1.9	718	9.3 ± 0.8	104
Consume weekly	39.9 ± 1.8	345	0.8 ± 0.5	12
Consume 5 days last week^§^	6.6 ± 1.3	84	0.4 ± 0.2	6
≥ 5 drinks any day^||^	38.6 ± 2.7	330	0.4 ± 0.2	7
				
Fruit & vegetables				
5+ servings/day	30.2 ± 2.1	243	26.5 ± 3.0	238
				
Physical activity^¶^				
Low (<600 MET-mins)	32.7 ± 5.3	281	40.4 ± 2.4	405
Moderate (600–2999 MET-mins)	16.7 ± 2.2	171	24.2 ± 1.4	247
High (3000+ MET-mins)	50.6 ± 5.3	452	35.4 ± 3.1	396

* Non-communicable diseases.

^† ^Standard errors.

^‡ ^Consume alcohol in the last 12 months.

^§ ^Consume alcohol on at least 5 days in the past 7 days.

^|| ^Consume 5 drinks or more on any single day in the last 7 days.

^¶ ^Moderate and/or vigorous activity in a typical week.

The results of pathophysiological measurements are presented in Table 3. The mean BMI was 21.2 (95%CI: 20.6–21.9) kg/m^2 ^for men and 21.5 (95%CI: 21.2–21.8) kg/m^2 ^for women. The proportions of excess body weight (BMI ≥ 25 kg/m^2^) for male and female participants in this sample were 11.1%, and 14.1%, respectively. Using the WHO recommended cut point for Asian populations, 23.6 (95% CI: 15.4–31.8) % of men and 31.8 (95%CI: 28.7–34.9) % of women in this sample were overweight or obese (BMI ≥ 23 kg/m^2^). The mean waist circumference was 75.0 (95%CI: 73.0–77.1) cm for men and 72.2 (95%CI: 71.2–73.3) cm for women. The proportion of abdominal obesity (waist circumference ≥ 90 cm for men or waist circumference ≥ 80 cm for women) was 7.7 (95%CI: 4.4–11.0) % for men, 17.8 (95%CI: 15.3–20.3) % for women, and 12.9 (95%CI: 10.0–15.8) % for both sexes combined. The mean SBP was 128.4 (95%CI: 126.5 – 130.4) mmHg for men and 120.1 (95% CI: 118.3–121.8) mmHg for women. The mean fasting BG was 3.62 (95%CI: 3.44–3.81) mmol/L for men and 3.65 (95%CI: 3.47–3.82) mmol/L for women. The mean fasting TC was 4.44 (95%CI: 4.30–4.58) mmol/L for men and 4.66 (95%CI: 4.56–4.75) mmol/L for women.

**Table 3:** Prevalence of pathophysiological risk factors for NCD* in Can Tho, 2005.

	Men (N = 910)		Women (N = 1066)	
		
	% ± SE^†^	n	% ± SE^†^	n
Body mass index				
<18.5 kg/m^2^	16.8 ± 2.8	174	17.4 ± 1.9	172
18.5–19.9 kg/m^2^	21.8 ± 2.1	201	18.6 ± 1.5	169
20.0–22.9 kg/m^2^	37.8 ± 2.4	327	32.2 ± 2.4	354
23.0–24.9 kg/m^2^	12.5 ± 2.4	112	17.7 ± 0.9	192
25.0–29.9 kg/m^2^	8.8 ± 2.1	83	12.6 ± 1.1	155
30+ kg/m^2^	2.3 ± 1.2	10	1.5 ± 0.4	22
				
Hypertension^‡^				
Yes	27.3 ± 2.5	320	16.2 ± 1.5	280
No	72.7 ± 2.5	590	83.8 ± 1.5	786
				
Blood glucose				
<6.1 mmol/l	99.0 ± 0.6	856	98.9 ± 0.2	1014
6.1–7.0 mmo/l	0.6 ± 0.5	5	0.3 ± 0.1	8
7.0+ mmol/l	0.4 ± 0.2	3	0.8 ± 0.2	16
				
Total cholesterol				
<5.2 mmol/l (200+ mg%)	85.5 ± 2.4	730	79.1 ± 2.1	770
5.2+ mmol/l (200+ mg%)	14.5 ± 2.4	136	20.9 ± 2.1	268

*Non-communicable diseases.

^† ^Standard errors

^‡ ^Systolic blood pressure ≥ 140 mmHg and/or diastolic blood pressure ≥ 90 mmHg or taking medication for hypertension.

Table 4 shows the estimates of pathophysiological risk factors in the four age groups sampled. More strongly for men than for women, mean levels of BMI (p < 0.001), and – adjusted for BMI – means levels of BG (p = 0.137) and TC (p < 0.001) increased with age. Mean levels of SBP adjusted for BMI also increased more strongly (p = 0.036) for women than for men, but from a lower level and did not overtake mean SBP of men even among 55–64 year olds. Adjusted also for the behavioural risk factors (smoking, alcohol, fruit and vegetable consumption and physical activity), the difference in trends remained stronger for women (SBP p = 0.031, BG, p = 0.009, TC p = 0.014).

**Table 4:** NCD* risk factor estimates by age group and sex in Can Tho, 2005.

	BMI^†^	SBP^‡^	BG^§^	TC^||^
	Mean(SE)	Mean(SE)	Mean(SE)	Mean(SE)
Men				
25–34	21.4(0.6)	126.0(1.7)	3.56(0.08)	4.39(0.09)
35–44	21.2(0.2)	125.0(1.2)	3.64(0.09)	4.37(0.06)
45–54	21.3(0.3)	134.3(1.3)	3.75(0.10)	4.57(0.08)
55–64	20.8(0.3)	140.0(1.5)	3.65(0.10)	4.66(0.06)
*p for trend*^¶^	*p = 0.543*	*p < 0.001*	*p = 0.015*	*p = 0.008*
				
Women				
25–34	20.5(0.2)	114.3(1.0)	3.49(0.10)	4.46(0.08)
35–44	21.4(0.3)	116.5(1.3)	3.61(0.10)	4.45(0.05)
45–54	22.9(0.3)	129.3(1.0)	3.87(0.09)	4.99(0.07)
55–64	22.6(0.3)	136.6(1.8)	3.93(0.12)	5.26(0.10)
*p for trend*^¶^	*p < 0.001*	*p < 0.001*	*p < 0.001*	*p < 0.001*

* Non-communicable diseases.

^† ^Body mass index.

^‡ ^Systolic blood pressure.

^§ ^Blood glucose.

^|| ^Total cholesterol.

^¶ ^p for trend adjusted for age.

**Figure 1:**
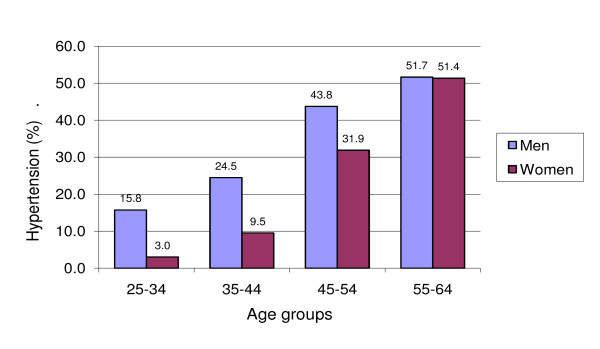
**Prevalence of hypertension by age group in Can Tho, 2005**.

More men (27.3%) than women (16.2%) were hypertensive (SBP ≥ 140 mmHg and/or DBP ≥ 90 mmHg, or taking medication for hypertension). Only 29.2% of hypertensive men, but 56.6% of hypertensive women, were aware of their condition. The prevalence of hypertension increased with age for both men (p < 0.001) and women (p < 0.001) (Figure 1). The age-standardised prevalence of hypertension was 26.7% for men and 15.9% for women.

**Figure 2:**
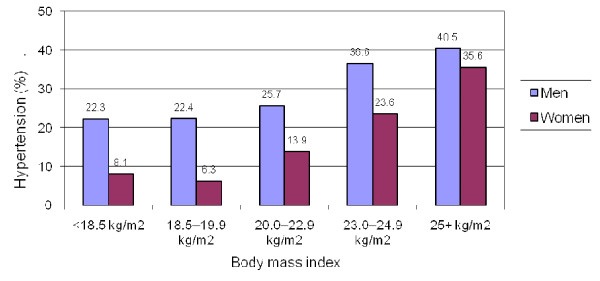
**Association between body mass index and hypertension in Can Tho, 2005**.

The associations between BMI and hypertension are presented in Figure 2. Hypertension prevalence increased linearly with BMI (p = 0.020 for men and p < 0.001 for women) after adjustment for age. For each unit of BMI increase, the odds of having hypertension increase by 11% (95%CI: 2–20%) for men and 17% (95%CI: 11–23%) for women. In each BMI category, there was a higher proportion of hypertensive men than women even after adjusting for age.

## Discussion

This is the first population survey using internationally standardised protocols to report the prevalence of risk factors for NCD in the Mekong Delta, Vietnam. Previous surveys on NCD have been conducted in Ha Noi and HCMC. There have been some data reported for the population of Bavi [5], an extremely poor district of Hanoi [15], but the findings from this study are unlikely to represent the risk profile of the population of the Mekong Delta where income is much higher and land holdings are much larger.

The first principal finding of this study was that older women in this population-based representative sample generally had an unfavourable NCD risk profile. The sex differences in SBP and TC persisted after adjustment for BMI. This has not been reported previously for Asian populations, but it mirrors reports for some Western populations that blood pressure and TC of men and women converge with advancing age [16-18]. For BG, we found a statistically significant stronger cross-sectional increase with age for women that was diminished by adjustment for BMI but strengthened by additional adjustment for behavioural risk factors. This cross-sectional pattern of increasing levels of BG with age for women, and higher levels for women at older ages, appears not to have been reported previously. Factors not measured in this survey, and which may account for the elevated risk among women, were hormonal status, saturated fat consumption and salt intake.

The second principal finding was the risk profile of this predominantly rural population of Vietnam was markedly different to that reported previously for the two major cities. The prevalence of raised BG (defined as capillary whole BG of at least 6.1 mmol/L) in our sample (men: 1.0%, women: 1.1%) are lower than prevalence estimates reported for the big city convenience samples: 2.7% for men and 2.6% for women aged 20–60 in HCMC in 2004 [8], 4.6% of men and 5.8% of women in the subset of participants aged 25–64 in another sample of HCMC population in 2001 [7], and 5.8% of 20–74 years old residents in Ha Noi in 2005 [4]. A possible explanation for these differences lies in the higher proportion of overweight and obesity observed in the big city surveys. The proportions of obese (BMI at least 23 kg/m^2^) participants in our sample (men 23.6%, women 31.8%) were lower than that reported for the Ha Noi sample (33.7%) [4], fewer participants in our survey (12.9%) than in the Ha Noi sample (17.5%) [4] had abdominal obesity (waist circumference ≥ 90 cm for men or waist circumference ≥ 80 cm for women). In the HCMC sample, 18.6% of participants had BMI at least 25 kg/m^2 ^but only 12.7% (men 11.1%, women 14.1%) of our participants exceeded this level [7]. In the rural sample of Ha Noi from Bavi, only 3.5% of participants had BMI at least 25 kg/m^2 ^[5]. Another possible explanation for the higher prevalence of raised BG in the big city samples is the lower levels of physical activity among the urban residents. There was 46.6% of men and 41.3% of women aged 25–64 in a HCMC sample in 2005 [9] classified as having a low level of physical activity compared to 32.7% of men and 40.4% of women in our samples.

In contrast to raised BG, the proportions of participants in our sample with hypertension (27% of men and 16% of women) exceed the prevalence estimates reported for the big city surveys. Hypertension was identified among 21% of men and 10% of women in a sample of 25–64 year olds from a single commune in Ha Noi in 2007 [3], 17.5% for the subset of 20–59 year old participants of a convenience sample from two districts of Ha Noi in 2005 [4], and 11% for men and 9% for women aged 20–60 in HCMC [8]. Our results are more similar to those reported for the extremely poor rural sample of Ha Noi from Bavi [5] in 2005 (24% for men and 14% for women aged 25–64) in which the prevalence of overweight and obesity was less than a third of that found this study. Dietary sodium intake has been linked with hypertension [19], and salt consumption may be higher in poor rural areas where it is used to add flavour to rice. No published study to date has measured dietary sodium levels in a Vietnamese population, however, and this contention remains unsupported. Alcohol has been shown to be associated with elevated blood pressure [20]. Our prevalence estimates of alcohol consumption are the only published Vietnamese data, and, therefore, no comparisons are possible. Tobacco smoking has been associated with elevated blood pressure in another study from this region [21]. The limited data on smoking prevalence in Vietnam show relatively minor variation through the country. In our sample, 67.8% of men and 1.1% of women were current smokers. In a HCMC sample of 20–60 year olds, 62.2% of men and 1.4% of women were current smokers in 2004 [8] and 62.9% of men and 0.6% of women were current smokers in the Bavi sample [5].

A key strength of this study was its use of a representative sample, with analysis done taking into account the complex survey design. The relatively high response proportion minimises the likelihood of selection bias, and the range and quantum of factors that were measured should be a good reflection of those factors in the Vietnamese population. The use of WHO standardised protocols, intensive training of data collection staff, pre-study testing of procedures, and the close supervision of staff during data collection, all highlight the attention that was paid to minimising avoidable sources of measurement error.

Limitations of this study need to be borne in mind. The STEPS methodology is designed to provide standardised information on key modifiable risk factors that can be measured in population-based surveys without resort to high technology instruments. It is not designed to measure total energy intake, dietary fat, dietary sodium, body fatness, or physical activity by objective methods such as accelerometry and pedometry. Information on these factors would have provided a more comprehensive picture of the relationships we studied. In addition, these cross-sectional data do not allow age-related differences in blood pressure, BG and TC to be attributed to ageing independently of cohort effects.

## Conclusion

This study provides the first NCD risk factor profile of people in the Mekong Delta of Southern Vietnam using internationally standardised methodology. Our findings for this predominantly rural sample differ from previous studies conducted in Ha Noi and HCMC, and suggest that it is inappropriate to generalise findings from the big-city surveys to the more than 80% of Vietnamese people who live outside the two commercial centres.

## Competing interests

The authors declare that they have no competing interests.

## Authors' contributions

All authors provided input into drafts and approved the final draft of the manuscript. In addition, PHL contributed to the design of the study and data acquisition; ABT contributed to the design of the study, data acquisition, data analyses and interpretation; LB contributed to the design of the study, data acquisition, data analyses and interpretation, and provide statistical expertise; TBN contributed to the design of the study and data acquisition; MDS contributed to data analyses and interpretation; RHG contributed to the design of the study; TD contributed to the design of the study. All authors read and approved the final version of the document.

## Pre-publication history

The pre-publication history for this paper can be accessed here:

http://www.biomedcentral.com/1471-2458/9/291/prepub
